# Chibby suppresses growth of human SW480 colon adenocarcinoma cells through inhibition of β-catenin signaling

**DOI:** 10.1186/1750-2187-7-6

**Published:** 2012-05-31

**Authors:** Victoria Fischer, Dex-Ann Brown-Grant, Feng-Qian Li

**Affiliations:** 1 Graduate Program in Molecular and Cellular Pharmacology, Stony Brook University, BST 7-186, Nicolls Rd, Stony Brook, NY, 11794-8651, USA; 2 Department of Pharmacological Sciences, Stony Brook University, BST 7-168, Nicolls Rd, Stony Brook, NY, 11794-8651, USA

**Keywords:** Chibby, Wnt/β-catenin signaling, Cyclin D1, Cell growth, Colon cancer

## Abstract

The canonical Wnt signaling pathway is crucial for embryonic development and adult tissue homeostasis. Activating mutations in the Wnt pathway are frequently associated with the pathogenesis of various types of cancer, particularly colon cancer. Upon Wnt stimulation, β-catenin plays a central role as a coactivator through direct interaction with Tcf/Lef transcription factors to stimulate target gene expression. We have previously shown that the evolutionarily conserved protein Chibby (Cby) physically binds to β-catenin to repress β-catenin-dependent gene activation by 1) competing with Tcf/Lef factors for binding to β-catenin and 2) facilitating nuclear export of β-catenin via interaction with 14-3-3 proteins. In this study, we employed human colon adenocarcinoma SW480 cells with high levels of endogenous β-catenin to address a potential tumor suppressor role of Cby. In SW480 stable cells expressing wild-type Cby (CbyWT), but not 14-3-3-binding- defective Cby mutant CbyS20A, a significant fraction of endogenous β-catenin was detected in the cytoplasm. Consistent with this, CbyWT-expressing cells showed low levels of β-catenin signaling activity, leading to reduced growth. Our results suggest that Cby, in collaboration with 14-3-3 proteins, can counteract oncogenic β-catenin signaling in colon cancer cells.

## Background

The canonical Wnt/β-catenin signaling pathway is highly conserved throughout evolution and plays diverse roles in embryonic development and adult homeostasis by regulating cell proliferation, cell fate decisions, as well as stem cell maintenance and self-renewal [1-4]. β-Catenin serves as a key transcriptional coactivator for transducing canonical Wnt signals from the cell surface to the nucleus. In the absence of Wnt ligands, cytoplasmic β-catenin becomes sequentially phosphorylated by casein kinase 1 (CK1) and glycogen synthase kinase 3 (GSK3) in the so-called “destruction complex” containing the tumor suppressors Axin and APC, and then targeted for ubiquitin-mediated proteasomal degradation [2,3,5]. Wnt binding to the Frizzled (Fz) receptors and LRP5/6 co-receptors leads to inhibition of β-catenin phosphorylation, resulting in stabilization of β-catenin protein. Subsequently, β-catenin translocates into the nucleus where it forms a complex with the Tcf/Lef transcription factors to stimulate expression of direct target genes such as cyclin D1 [6-8].

Sustained activation of Wnt/β-catenin signaling, due to loss-of-function mutations in APC or Axin or gain-of-function mutations in β-catenin, has been linked to various human malignancies including melanoma and colon and hepatocellular carcinomas [1,7,9,10]. Notably, greater than 70% of colon cancers exhibit elevated Wnt/β-catenin signaling activity. Mutations in APC or Axin compromise their function within the β-catenin destruction complex, whereas oncogenic mutations in the N-terminal phosphorylation domain of β-catenin block its degradation via the ubiquitin-proteasome pathway. In all cases, the common outcome is the stabilization and nuclear accumulation of the central player β-catenin and subsequent activation of its target genes. Accordingly, the Wnt/β-catenin pathway has gained recognition as an enticing molecular target for therapeutics of human cancers [7,11,12].

Chibby (Cby) was originally identified as a β-catenin binding partner using the C- terminal transactivation domain of β-catenin as bait in a yeast Ras recruitment system [13]. Cby is a 14.5-kDa protein evolutionarily conserved from fly to human. We have previously reported that Cby acts as a Wnt/β-catenin antagonist through two distinct molecular mechanisms [14,15]. It competes with Tcf/Lef factors for β-catenin binding in the nucleus. Cby also interacts with 14-3-3 chaperones to export β-catenin out of the nucleus. 14-3-3 proteins specifically recognize serine 20 within the 14-3-3-binding motif of Cby. More recently, we have demonstrated that Cby harbors functional nuclear localization signal (NLS) and nuclear export signal (NES) motifs and constitutively shuttles between nucleus and cytoplasm [16]. Direct interaction of 14-3-3 proteins with Cby facilitates Cby binding to the CRM1 export receptor, while inhibiting Cby binding to the nuclear import receptor importin-α, thereby promoting cytoplasmic compartmentalization of Cby and β-catenin.

It has been reported that Cby expression is significantly down-regulated in pediatric ependymomas [17] and colon cancer cell lines [18]. However, the tumor suppressive properties of Cby remain largely uncharacterized. In this study, we evaluated whether Cby suppresses β-catenin-dependent signaling activity and growth of the human colon adenocarcinoma cell line SW480 in which APC is mutated, causing stabilization and nuclear accumulation of β-catenin [19,20]. We show that overexpression of Cby in SW480 cells causes a dramatic shift in distribution of β-catenin towards the cytoplasm, thereby repressing β-catenin-mediated gene activation. Furthermore, stable expression of Cby inhibited SW480 cell growth. Notably, these effects are, at least in part, exerted through interaction with 14-3-3 proteins since stable expression of 14-3-3-binding- defective Cby mutant CbyS20A has no significant influences on β-catenin signaling and cell growth. Taken together, these findings support a role for Cby in the control of colon tumorigenesis.

## Results

### Ectopic expression of Cby represses endogenous β-catenin signaling in SW480 colon cancer cells

Previously, we reported that Cby serves as a β-catenin signaling antagonist by competing with Tcf/Lef factors for β-catenin binding and facilitating nuclear export of β-catenin in concert with 14-3-3 proteins [14-16]. To investigate whether Cby influences endogenous β-catenin signaling, we turned to the human colon adenocarcinoma SW480 cell line. It is well established that the canonical Wnt/β-catenin signaling pathway is aberrantly activated in most colon cancers. The SW480 cells carry truncating mutations in the *APC* gene and consequently accumulate nuclear β-catenin, leading to constitutive activation of β-catenin signaling [19,20]. Interestingly, Cby expression in these cells is down-regulated due to hypermethylation of the *cby* promoter region [18], providing a useful model system to study Cby function.

**Figure 1:**
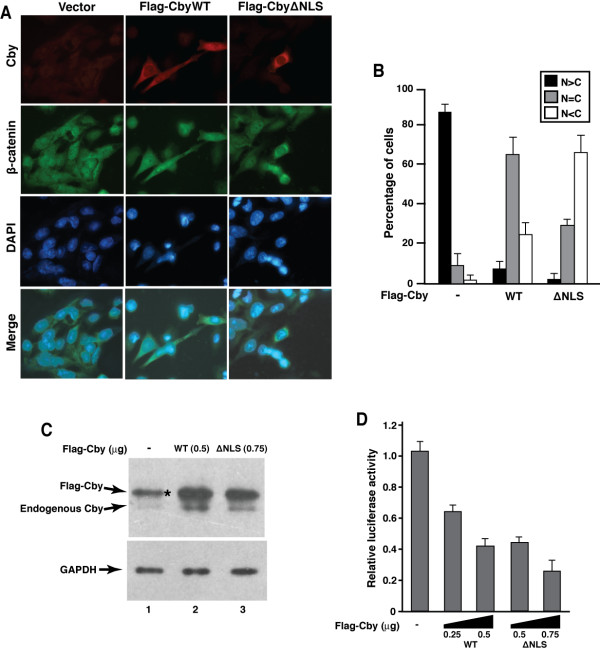
**Forced expression of Cby in SW480 colon cancer cells promotes translocation of nuclear β-catenin towards the cytoplasm, resulting in inhibition of endogenous β-catenin signaling.(A)** SW480 cells were transiently transfected with a control empty vector or an expression plasmid for Flag-tagged CbyWT or Cby∆NLS, and doubly immunostained with anti-Cby (red) and anti-β-catenin (green) antibodies. Nuclei were stained with DAPI. A merged image of β-catenin and DAPI is also shown. **(B)** Quantitative analysis of the results in (A). The subcellular localization of endogenous β- catenin was scored as follows: N>C, predominantly nuclear; N=C, evenly distributed between the nucleus and cytoplasm; N<C, predominantly cytoplasmic. Error bars represent the means ± SD of three independent experiments. For cells transfected with Cby plasmids, β-catenin localization was scored only in those expressing ectopic Cby. **(C)** Western blot analysis of Cby expression in SW480 cells using anti-Cby antibody. Note that, to compensate protein levels, higher amounts of DNA for Cby∆NLS were used for transfection. The anti-Cby antibody detected both exogenous and endogenous proteins. An asterisk indicates a non-specific band that overlaps with Flag-Cby. GAPDH was used to confirm equal loading. **(D)** The ability of Cby to repress endogenous β- catenin signaling was tested by TopFlash assays. SW480 cells were transfected with 100 ng of TopFlash luciferase reporter and the indicated amounts of a Flag-tagged Cby expression plasmid. Luciferase activity was measured 24 h post-transfection, and normalized to Renilla luciferase activity used as an internal control. All transfections were carried out in triplicates and the means ± SD are shown.

We first assessed if ectopic expression of Cby affects β-catenin subcellular distribution in SW480 cells. In agreement with prior reports [21,22], endogenous β-catenin was detected in the nucleus of the vast majority of cells (Figure 1). Transient expression of wild-type Cby (CbyWT) led to a significant shift in β-catenin localization towards the cytoplasm. Next, we examined the effect of a Cby mutant lacking the functional C- terminal NLS (Cby∆NLS) since this mutant is exclusively cytoplasmic and able to retain exogenous β-catenin in the cytoplasmic compartment [16]. Ectopic expression of Cby∆NLS resulted in even more profound cytoplasmic enrichment of β-catenin where they colocalized (Figure 1A and B).

Western blot analysis using anti-Cby antibody revealed that as previously noted at mRNA levels [18], endogenous Cby protein was present at low levels in non-transfected SW480 cells (Figure 1C, lane 1). Upon transient transfection, expression levels of Cby∆NLS were lower compared to those of CbyWT. We therefore increased the amount of DNA for the mutant for the subsequent β-catenin-dependent luciferase reporter (TopFlash) assays. SW480 cells exhibit high levels of TopFlash activity resulting from constitutive activation of β-catenin signaling (Figure 1D). Ectopic expression of CbyWT repressed TopFlash activity in a dose-dependent manner. In accordance with our previous observations in HEK293T cells [16], Cby∆NLS exerted slightly more potent inhibitory effects through efficient sequestration of β-catenin in the cytoplasm . Taken together, our results indicate that Cby negatively modulates endogenous β-catenin signaling in SW480 cells.

### Stable expression of Cby suppresses colon cancer cell growth

**Figure 2:**
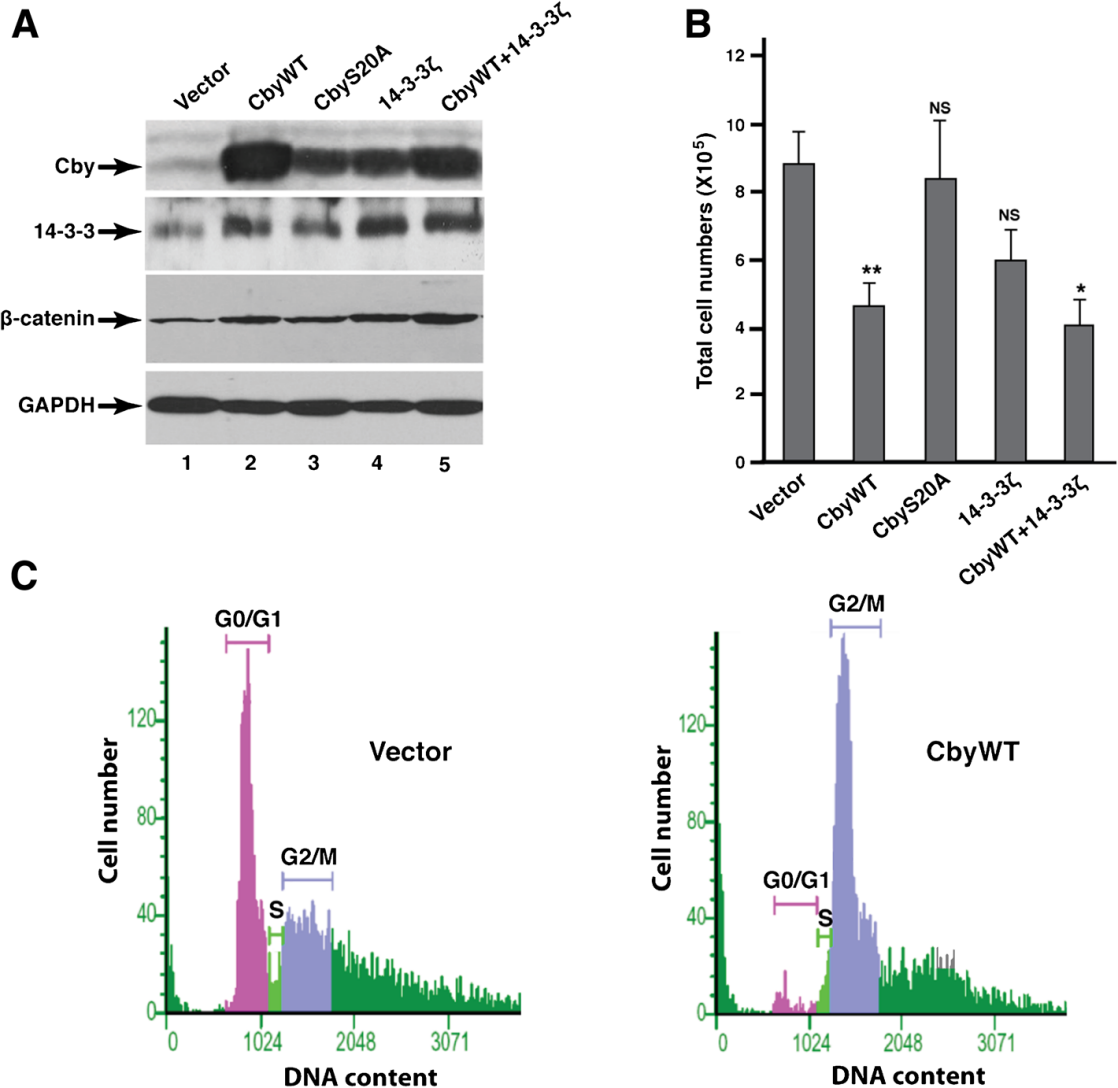
**Stable expression of Cby reduces SW480 cell growth.(A)** Stable SW480 cell lines expressing CbyWT, CbyS20A, 14-3-3ζ or both Cby and 14-3-3ζ, and vector- control cells were established, and the cell lysates were analyzed by western blotting using antibodies against Cby, 14-3-3, β-catenin and GAPDH. **(B)** The stable SW480 cell lines were seeded on 24-well plates (10^5^cells/well), and total cell numbers were counted 5 days later. The data are the means ± SE of triplicate samples and representative of three independent experiments. Student’s *t*-test; *P < 0.05, **P < 0.01, NS = not significant when compared with vector-control cells. **(C)** Cell cycle analysis of vector-control and CbyWT-expressing cells. Following propidium iodine staining, the DNA content of individual cells was measured by flow cytometry. The data shown are from one representative experiment out of three.

It was shown that the proliferation of colon cancer cells largely depends on the continuous presence of β-catenin signaling [23,24]. Having established that Cby interferes with β-catenin signaling activity in SW480 cells, we investigated whether Cby influences their growth. To this end, we generated stable cell lines by infecting SW480 cells with a retrovirus carrying CbyWT, 14-3-3-binding-defective CbyS20A, 14-3-3ζ or both Cby and 14-3-3ζ along with a control retrovirus. The efficient expression of the proteins was confirmed by immunoblotting using anti-Cby and anti-14-3-3 antibodies (Figure 2A). Worthy of note, stable expression of CbyWT substantially stabilized endogenous 14-3-3 proteins, reaching near exogenous expression levels (compare lanes 2 and 4). Similarly, stable expression of 14-3-3ζ resulted in an increase in endogenous Cby protein levels (compare lanes 1 and 4).

As shown in Figure 2B, stable expression of CbyWT significantly suppressed growth of SW480 cells. Coexpression with 14-3-3ζ showed no further influence on their growth. This is most likely explained by the fact that stable expression of CbyWT increases levels of endogenous 14-3-3 protein (Figure 2A). In contrast, CbyS20A displayed no obvious effect, suggesting that Cby-14-3-3 interactions are crucial for suppression of SW480 cell growth. Stable expression of 14-3-3ζ alone showed a tendency to slow cell growth but it was not statistically significant, implying that efficient growth inhibition requires relatively high expression levels of both Cby and 14-3-3 proteins. Cell cycle analysis by flow cytometry showed that there is a marked increase in the G2/M population with a concomitant decrease in the G0/G1 population in CbyWT-overexpressing cells in comparison with vector-control cells (Figure 2C). Collectively, these observations suggest that Cby, in cooperation with 14-3-3 proteins, attenuates colon cancer cell growth by inducing a G2/M cell-cycle arrest.

### Stable expression of Cby reduces nuclear β-catenin levels in SW480 cells

**Figure 3:**
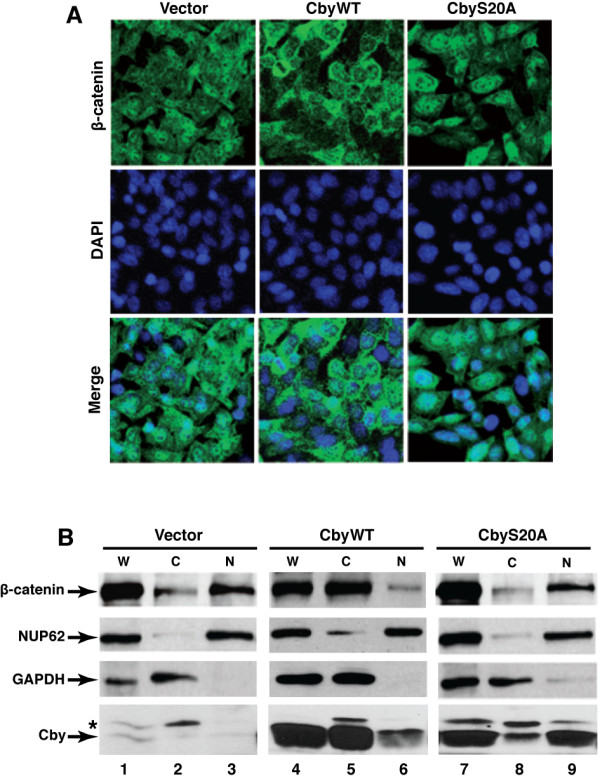
**Cby facilitates nuclear export of endogenous β-catenin.(A)** SW480 cells stably expressing CbyWT or CbyS20A and control cells were fixed and subjected to immunofluorescence staining of endogenous β-catenin (green). Nuclei were visualized with DAPI. **(B)** Whole-cell (W), nuclear (N) and cytoplasmic **(C)** extracts were prepared from the indicated SW480 stable cells, and β-catenin levels were analyzed by western blotting. The relative purity of nuclear and cytoplasmic fractions was evaluated by probing for nucleoporin p62 (NUP62) and GAPDH, respectively.

We previously reported that Cby is a nuclear-cytoplasmic shuttling factor and acts with 14-3-3 proteins to promote nuclear export of β-catenin [14,16]. Thus, we examined whether the growth-suppressive effect of Cby on SW480 cells is mediated in part by changes in β-catenin subcellular localization using immunofluorescence microscopy. In control SW480 cells, β-catenin was predominantly detected in the nucleus (Figure 3A). However, in stable cells expressing CbyWT but not CbyS20A, there was a shift in β-catenin localization towards the cytoplasm (Figure 3A).

To confirm these results, we performed subcellular fractionation of SW480 cell lysates in order to assess relative β-catenin levels in nuclear and cytoplasmic compartments. Consistent with our immunofluorescence staining data, stable expression of CbyWT led to a cytoplasmic enrichment of β-catenin with a concomitant reduction in its nuclear levels (Figure 3B, lanes 5 and 6). CbyS20A-expressing cells exhibited no major changes in β-catenin distribution (lanes 8 and 9). It is also noteworthy that CbyWT showed marked enrichment in the cytoplasmic fraction (lanes 5 and 6), whereas CbyS20A was more abundant in the nuclear fraction (lanes 8 and 9). This further confirms our prior data demonstrating that association of 14-3-3 with Cby at serine 20 is responsible for cytoplasmic sequestration of Cby [14,16]. These findings suggest that Cby, in cooperation with 14-3-3 proteins, shuttles β-catenin out of the nucleus into the cytoplasm, leading to reduced cancer cell growth.

### Stable expression of Cby inhibits β-catenin signaling in colon cancer cells

**Figure 4:**
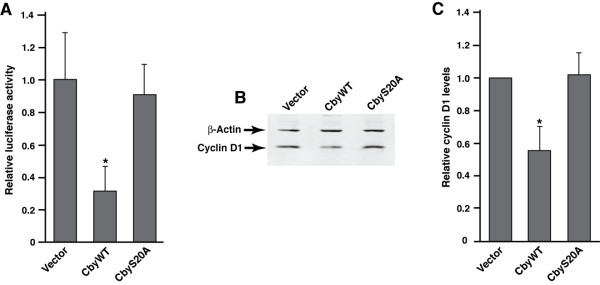
**β-Catenin signaling activity is attenuated in SW480 cells stably expressing Cby.(A)** CbyWT, but not CbyS20A, represses TopFlash activation. SW480 stable cells were transfected in 24-well plates with 25 ng of TopFlash or mutant FopFlash reporter as well as a Renilla luciferase plasmid to normalize transfection efficiency. Luciferase activities were measured 48 h post-transfection, normalized and corrected for background by subtraction of FopFlash values from corresponding TopFlash values. Each transfection was performed in triplicate and repeated at least three times. Shown are the means ± SD from one representative experiment. Student’s *t*-test; *P < 0.05 when compared with vector-control cells. **(B)** CbyWT, but not CbyS20A, inhibits cyclin D1 expression. Whole-cell lysates from the indicated SW480 stable cells were subjected to western blot analysis for cyclin D1 and beta-actin as a loading control. **(C)** Quantification of cyclin D1 expression levels. The intensity of cyclin D1 bands from western blots was quantified using the NIH Image software and normalized to that of beta-actin bands. Data are the mean band intensities ± SD from at least three independent experiments. Student’s *t*-test; *P < 0.05 when compared with vector-control cells.

To investigate whether the compromised growth of CbyWT-expressing cells is accompanied by a reduction in the transcriptional output of β-catenin, we conducted TopFlash reporter assays. As expected, we observed that β-catenin signaling was markedly reduced in SW480 stable cells expressing CbyWT but not CbyS20A (Figure 4A). Cyclin D1 is a direct target for the β-catenin/Tcf complex, and plays a crucial role in proliferation of colon cancer cells [24,25]. We found that expression of cyclin D1 was reduced in SW480 cells expressing CbyWT but not CbyS20A (Figure 4B and C). Taken together, our findings suggest that Cby, in cooperation with 14-3-3 proteins, suppresses colon cancer cell growth by modulating subcellular distribution and signaling activity of endogenous β-catenin.

## Discussion

It has been established that the canonical Wnt/β-catenin signaling pathway is aberrantly activated in the vast majority of colon cancers and to a lesser extent in other tumor types. Most common mutations occur in the *APC* or *β-catenin* gene, which ultimately results in stabilization and nuclear accumulation of β-catenin in cancer cells. Thus, a deeper understanding of β-catenin regulation is important for the development of effective cancer therapies.

The evolutionarily conserved Cby protein binds to the C-terminal transactivation domain of the β-catenin oncoprotein and acts as a potent repressor, thereby attenuating canonical Wnt signaling [13,14,16,26,27]. Cby mRNA levels have been shown to be down-regulated in various colon cancer cell lines including SW480 cells due to promoter hypermethylation [18]. However, a potential tumor suppressor function of Cby remains largely unknown. The goal of this research was to determine whether Cby, in conjunction with 14-3-3, regulates growth of SW480 colon cancer cells by controlling β-catenin localization and signaling activity. Consistent with our hypothesis, stable expression of Cby suppresses SW480 cell growth. In these cells, β-catenin is predominantly found in the cytoplasm and its signaling activity is significantly reduced. This effect of Cby is most likely to be mediated by 14-3-3 proteins since CbyS20A defective in 14-3-3 binding has no significant effect on cell growth and β-catenin signaling. Manipulation of Cby function might therefore provide a novel means for the therapeutic intervention of Wnt/β-catenin-driven tumors.

Interestingly, stable expression of Cby causes a dramatic increase in G2/M phase cells (Figure 2C). In support of this, β-catenin has been implicated in the control of cell cycle at G2/M [28,29], and small molecule inhibitors of β-catenin signaling (quercetin and NC043) have been shown to block the cell cycle of SW480 cells at the G2/M phase [30,31]. These findings concur with the notion that reduced SW480 cell proliferation by Cby overexpression is primarily attributable to a G2/M cell-cycle arrest. At present, the exact fate of Cby-14-3-3-bound β-catenin is unknown. However, we speculate that the Cby-14-3-3-β-catenin complex might be protected from degradation pathways and remains stable in the cytoplasm. This may serve as a reservoir of signaling competent β-catenin that is readily available for release in response to upstream signals.

Our findings are in good agreement with the idea that Cby may function as a tumor suppressor. Interestingly, deletion and epigenetic silencing of the *Cby* gene was detected in over 60% of pediatric ependymomas [17]. However, it remains unclear whether Wnt/β- catenin signaling is up-regulated in ependymomas. On the other hand, no mutations or expression changes of the *Cby* gene have been reported in colon cancer [18,32] and Wilms tumors [33]. Nonetheless, it is possible that alterations of Cby function might occur through changes in posttranslational modifications that affect protein stability and subcellular distribution. Clearly, further experiments are required to define the tumor suppressor role of Cby in human cancer.

## Methods

### Expression constructs

The expression plasmids for Flag-tagged wild-type Cby (CbyWT) and CbyΔNLS have been described previously [14,16]. For generation of SW480 stable cell lines, Cby and 14-3-3ζ cDNAs were amplified by PCR and subcloned into the retroviral vector pQCXIP (puromycin resistant; Clontech). Additionally, a CbyWT cDNA was subcloned into pLXIN (neomycin resistant; Clontech) for establishment of the double-stable cell line with 14-3-3ζ. The sequence of all expression vectors was confirmed by DNA sequencing.

### Cell culture, transfection and viral infection

SW480 cells were purchased from ATCC and propagated in DMEM with 10% FBS and 100 U/ml penicillin-streptomycin. For transient transfection, cells were seeded onto 6- or 12-well tissue culture dishes, cultured overnight, and then transfected using Lipofectamine 2000 (Invitrogen) or Expressfect (Denville Scientific, Inc.) according to the manufacturer’s instructions. Empty vector was added to adjust the total amount of DNA to be the same in each transfection. For establishing stable cell lines, SW480 cells were infected with retroviruses bearing CbyWT, CbyS20A and 14-3-3ζ individually or CbyWT and 14-3-3ζ in combination, followed by selection of pools of cells with either 2.5 μg/ml of puromycin (Invitrogen) alone or both puromycin and 500 μg/ml of G418 (Invitrogen) as described previously [26].

### Immunofluorescence microscopy

Cells were seeded at 5x10^5^ cells/well onto cover slips in 12-well dishes and allowed to adhere and proliferate for 48 h. Cells were then fixed with methanol/acetone (1:1, v/v), incubated with anti-β-catenin antibody (1:1000; mouse monoclonal; BD Transduction Laboratories) and anti-Cby antibody (1:1000; rabbit polyclonal; [13]) in 1% BSA and 0.1% Triton X-100 in PBS, washed and incubated with DyLight 488-conjugated goat anti-mouse IgG and DyLight 549-conjugated goat anti-rabbit IgG secondary antibodies (1:500; Jackson ImmunoResearch Laboratories). Cells were counterstained with DAPI and mounted onto glass slides. Images were taken on a Zeiss LSM510 confocal microscope and processed with Adobe Photoshop for brightness and contrast. To quantify subcellular localization of β-catenin, independent transfections were performed at least three times and a minimum of 100 cells were counted for each transfection as described previously [14,16].

### Subcellular fractionation and immunoblotting

Whole cell lysates were prepared from subconfluent cells and fractionated as described [34]. Protein concentration was determined using the DC protein assay kit (Bio-Rad). Equal amounts of nuclear and cytoplasmic fractions were treated with concanavalin A- Sepharose (Sigma), using 7 μg concanavalin A/μg total protein, at 4°C for 3 h. The resultant supernatants were separated by SDS-PAGE, and subjected to western blotting. The primary antibodies used were as follows: rabbit anti-Cby [13]; rabbit anti-pan-14-3-3 (Santa Cruz Biotechnology); mouse anti-GAPDH (BioDesign International); rabbit anti- cyclin D1 (Epitomics); and mouse anti-β-catenin and mouse anti-nucleoporin p62 (BD Transduction Laboratories). HRP-conjugated secondary antibodies were purchased from Jackson immunoResearch Laboratories.

### TopFlash luciferase reporter assays

SW480 cells were seeded onto 12- or 24-well plates, and 24 h later, cells were transfected with the appropriate combinations of plasmids in triplicate. A Renilla luciferase (pRL- TK) was cotransfected to normalize transfection efficiency. Luciferase activity was measured 24 to 48 h post-transfection using the Dual-Luciferase Reporter Assay System (Promega) with a luminometer (Berthold Technologies) as described previously [14,16,27].

### Cell growth and cell cycle assays

Cells were plated in triplicate at 10^5^cells/well in 24-well plates. On day 5, cells were trypsinized, diluted, stained with 0.4% trypan blue (Sigma), and live cells were counted using a hemocytometer. For cell cycle analysis, cells were seeded at 5x10^5^ cells/well in 12-well plates and allowed to proliferate for 3 days. The cells were then harvested by trypsinization, fixed in ice-cold 70% ethanol overnight, and stained with propidium iodine (Sigma). The DNA content was measured using a Guava EasyCyte Plus flow cytometer (Millipore).

### Statistical analysis

Statistical significance was calculated by the unpaired Student’s *t-*test using Microsoft Excel. Data are presented as means ± SE or SD as indicated, and *P* values of <0.05 were considered statistically significant.

## Competing interests

The authors declare that they have no competing interests.

## Authors’ contributions

FQL designed the experiments. VF performed the experiments. DABG assisted VF with the TopFlash and immunofluorescence microscopy experiments. F-QL and VF wrote the manuscript. All authors have read and approved the final manuscript
